# Significant Changes in Cytoplasmic Amino Acid Composition Occur in the Transition between Mid-Exponential and Stationary Phases of Growth of *Staphylococcus aureus*: An Example of Adaptive Homeostasis in Response to Nutrient Limitations

**DOI:** 10.3390/microorganisms11010147

**Published:** 2023-01-06

**Authors:** Mousa Alreshidi, Hugh Dunstan, Margaret MacDonald, Mohd Saeed, Salem Elkahoui, Tim Roberts

**Affiliations:** 1 Department of Biology, College of Science, University of Ha’il, Ha’il 2440, Saudi Arabia; 2 Molecular Diagnostic and Personalized Therapeutics Unit, University of Ha’il, Ha’il 2440, Saudi Arabia; 3 InnovAAte Pty Ltd., 45 Hunter Street, Newcastle, NSW 2300, Australia; 4 Pathogenic Microbiology Laboratory, Faculty of Science, School of Environmental and Life Sciences, University Drive, Newcastle, NSW 2308, Australia

**Keywords:** *S. aureus*, stationary phase, adaptation metabolism, amino acids

## Abstract

The bacterial pathogen *Staphylococcus aureus* causes a wide range of infections that result in high morbidity and mortality rates worldwide. *S. aureus* is known for its capacity to survive harsh environments between hosts and certain strains are very efficient as opportunistic pathogens. It is important to understand their capacities for metabolic adaptation in response to changing environmental conditions. This investigation aimed to explore the alterations in the amino acid compositions of the cytoplasm as nutrients became limiting during the growth of *S. aureus*. Cells were grown under optimal growth conditions and harvested at the mid-exponential and stationary phases of growth and then extracted for the analyses of amino acids in the cytoplasm. The analyses revealed that the stationary phase cells had a significantly higher concentration of total cytoplasmic amino acids compared with cells at the mid-exponential phase and displayed substantial alterations in amino acid composition. Aspartic acid was the major amino acid in the stationary phase cells, whereas glutamic acid was the most abundant in the mid-exponential cells. The glutamic acid was reduced by 47% of its original value when the growth was extended to the stationary phase. Interestingly, certain amino acids were either absent or present depending on the phase of growth. These outcomes are in line with the premise that bacterial cells of *S. aureus* transition into a different form of metabolic homeostasis in the shift between the exponential and stationary phases of growth, as nutrients become depleted and waste products accumulate in the external medium. The ability of *S. aureus* to continually and promptly adapt to differences within growth phases may represent an essential strategy assisting its virulence as a successful opportunistic pathogen to establish infections. An understanding of the switch mechanisms controlling these obvious alterations in amino acids through the growth/life cycle of this virulent pathogen may provide novel clinical strategies to battle infection.

## 1. Introduction

*Staphylococcus aureus* poses a serious threat to public health and leads to 20,000 deaths per year in the United Sates alone [1]. Diseases associated with Staphylococcus spp. can result in high morbidity and mortality and thus present a high risk to both animals and humans alike [2]. The problem is exacerbated by methicillin-resistant *S. aureus* (MRSA), which is associated with 60% higher mortality compared with antibiotic-susceptible *S. aureus* [1]. *S. aureus* has arisen as a very effective pathogen because of its ability to quickly acclimatize to changes in the environmental parameters and its great array of core virulence factors [3,4,5]. These characteristics enable *S. aureus* to induce infections in a wide variety of tissues, facilitated by various routes of entry and causing a myriad of diverse diseases in both hospital and community settings. 

Microorganisms are mostly live in the stationary phase in their normal environments [6]. In reality, microbes live in environments that are sparse in nutrients and are surrounded by physical and chemical stresses that lead to unbalanced growth [7]. In addition, the transmission of staphylococcal infections in hospital occurs under limiting nutrients and stressful conditions. All of these events would induce significant alterations at the metabolic level [8]. Such alterations are similar to those observed during the transition into stationary phase in laboratory studies [7]. Investigations varying the nutrient supply to *E. coli* in a single-cell experimental system showed that these cells could maintain a relatively constant rate of protein synthesis for several days [6]. Determining how a clinical isolate of *S. aureus* adjusts its cytoplasmic amino acid composition in response to limited nutrients in the stationary phase is an essential key that might lead to better clinical performs for controlling the spread of *S. aureus* infections inside clinical settings [6,9]. Bacterial cells often induce biofilm formation during the stationary phase, which is regulated by quorum sensing [10], and are more resilient to disinfectants, antibiotics and detergents utilized in food manufacturing [11]. Bacteria produce the significant expression of virulence factors in the stationary phase [12,13]. Stationary phase cells are physiologically comparable to biofilm bacteria [7]. One key shift is the development of persister cells and small colony variants formed during the stationary phase, in biofilm and as a result of changes in growth conditions, including changes in pH, temperature and osmotic pressure [3,7,14].

Amino acid metabolites are crucial elements of the bacterial metabolism due to their wide involvements in many biochemical processes, including the synthesis of proteins, cell wall peptidoglycan and other components used to make nucleic acids and complex lipids, while certain amino acids are readily utilized for energy production and as osmoprotectants [15]. It was hypothesized that a clinical strain of *S. aureus* would adjust its amino acid metabolites to acquire ideal metabolic homeostasis and thus improve survivability during the transition to the stationary phase. This study aimed to ascertain the changes associated with amino acid homeostasis in *S. aureus* as the cells transition from the mid-exponential phase to the stationary phase, where nutrients have become limiting in the external medium, and metabolic waste products have accumulated.

## 2. Materials and Methods

### 2.1. Bacterial Strain

A clinical isolate of *S. aureus* was used in this study [16]. The strain used in the current investigation was a clinical isolate of *S. aureus* from patients that had been suffering from chronic muscle pain [17]. This isolate has been used in subsequent studies to measure metabolic and proteomic changes to environmental stimuli [4,5,13,18]. This isolate was plated in blood plate agar and its identity was frequently checked using the API^®^ Staph identification system, and was also determined by polymerase chain reaction (PCR). 

### 2.2. Media and Growth Conditions

An overnight culture was made by taking a colony of *S. aureus* from horse blood agar and inoculating it in 100 mL tryptic soy broth (TSB) (Oxoid Ltd-Melbourne, Australia), which was then placed on a shaker at 37 °C for almost 18 h to be used for the study experiments. The cultures of the experiments were conducted by taking 5 mL of overnight culture with 95 mL of TSB culture media (OD_600_ = 0.1 ≈ 10^8^ CFU/mL), which were then grown at 37 °C with constant agitation (120 rpm) to either the mid-exponential phase or stationary phase. Bacterial growth in TSB was determined by measuring the absorbance at 600 nm, which enabled the rapid assessment of the stage of growth of a culture. The broth cultures could then be collected at either the mid-exponential phase or stationary phase, as defined by prior growth curve experimental data. The experiments were repeated several separate times to optimize statistical assessment and ensure the reproducibility of the experiments. Cultures were then harvested by centrifugation at 6000× *g* for 25 min and collected cells were washed with phosphate-buffered saline (PBS). Washed cells were directly quenched using liquid nitrogen, freeze-dried and subsequently extracted for metabolic profiling analysis.

### 2.3. Analyses of Amino Acid Compositions

Approximately 10 mg of lyophilized cells were used for the extraction of cytoplasmic metabolites. Lyophilized cells were mixed with extraction buffer (1:1 ice cold methanol/water), vortexed, placed in liquid nitrogen and kept at −20 °C for 30 min to let the thawing process occur slowly. The contents of the solution were then separated by the centrifugation method to eliminate cell debris. The supernatant enclosing the metabolites was transferred into new 50 mL falcon tubes and placed in a drier vacuum to eradicate the methanol/water residues. Cytoplasmic amino acids were then extracted using EZ:faast kit (Phenomenex^®^ EZ: faast™) and subsequently applied to Agilent Gas Chromatography–Flame Ionization (GC-FID) (Hewlett Packard HP 6890 series) as earlier described [18].

### 2.4. Processing and Statistical Analysis of Amino Acid Data

GC-FID data were analyzed using MetaboAnalyst 5.0, which were firstly normalized by sum, log transformation, and auto-scaling methods. The differences in amino acids between the mid-exponential phase and stationary samples was identified using Volcano analysis (*p* < 0.01 and fold change < 2 was considered to be statistically significant). Partial least squares-discriminant analysis (PLS-DA) and principal component analysis (PCA) were applied to determine the general differences between the mid-exponential and stationary samples. Cross-validation was applied to assess the validity of the PLS-DA model. Significant variables (amino acids) contributing to the separation of mid-exponential and stationary phase amino acids in the PLS-DA were determined using the variable importance in the projection (VIP) score. Metabolic pathway analysis was conducted by submitting metabolite data to the MetabolAnalyst website [19]. The metabolite names were validated using KEGG and pubChem databases. The biochemical pathway database of *Staphylococcus aureus* N315 was chosen for pathway analysis.

## 3. Results

**Figure 1:**
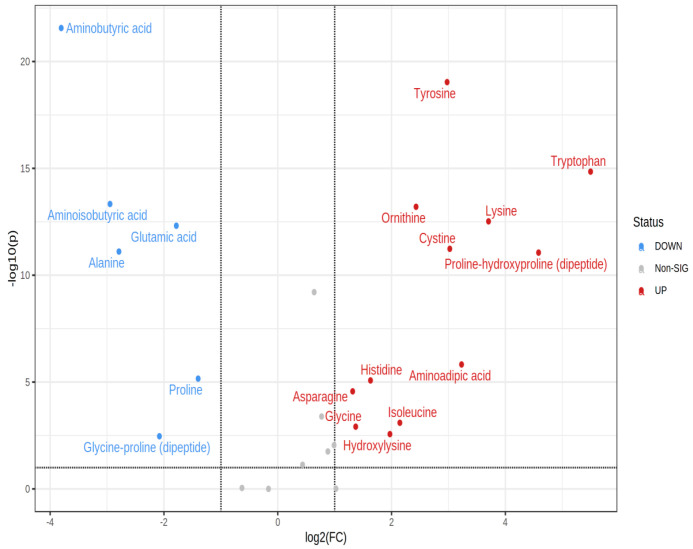
Volcano plot displaying increased (red) and decreased (blue) amino acids in cells grown to the stationary phase in comparison with the mid-exponential phase. Changes considered significant are those with fold change > |2| and *p* < 0.01.

Amino acid analyses of cells grown under optimal growth conditions revealed that eighteen amino acids were found to be altered at the exponential phase in comparison with the stationary phase (Table 1 and Figure 1). The amino acid concentration in the bacterial cells harvested at either the mid-exponential phase or stationary phase is shown in Table 1. Altered amino acids with at least a 2-fold change are shown in Figure 1. The concentration of total amino acids in the cytoplasm was increased by approximately 40% following extended growth to the stationary phase. This was mainly attributed to significant increases in lysine, histidine, and aspartic acid. In contrast, the most abundant amino acid in the mid-exponential cells was glutamic acid, but its levels dropped to 47% of the original level following extended growth to the stationary phase, where aspartic acid became the most abundant cytoplasmic amino acid. Aminobutyric acid was clearly present in the cytoplasm of the cells harvested at the mid-exponential phase but was virtually absent in stationary phase cells. Conversely, α-aminoadipic acid, hydroxylysine, and cystine were not detected in any samples at the exponential phase but were detected in cells at the stationary phase. 

**Figure 2:**
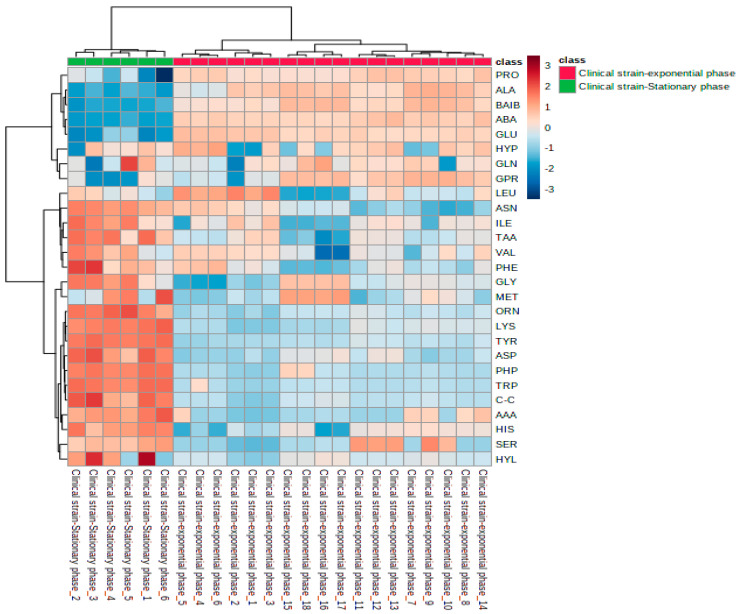
Heatmap analysis of amino acids extracted from cells harvested at the mid-exponential and stationary phases. Rows represent amino acids, and columns represent the replicates for each growth phase. The different colors of the heatmap depict the relative level of each amino acid between the cells grown to the mid-exponential and stationary phases. Red and blue colors denote higher or lower concentrations, respectively, of amino acid metabolites.

Heatmaps and the hierarchical clustering of the cytoplasmic amino acid dataset (Figure 2) provide a visual representation of the lower and higher abundance amino acids in each replicate of the cells harvested at the mid-exponential and stationary phases, where the individual concentrations of each amino acid are indicated. The clusters of the heatmap were processed according to Ward clustering method and the Euclidean distance was used for measuring similarity. This statistical technique revealed that the different growth phases clustered into two groups on the basis of their amino acid profiles. Heatmap analysis demonstrated that a decrease in ornithine, lysine, tyrosine, proline-hydroxyproline (dipeptide), tryptophan and cystine was common to all samples harvested at the mid-exponential phase in comparison with the samples analyzed at the stationary phase. Conversely, there was a consistent increase in proline, alanine, ß-aminoisobutyric acid, α-aminobutyric acid and glutamic acid in all replicates analyzed at the mid-exponential phase in contrast with the replicates analyzed at the stationary phase.

**Figure 3:**
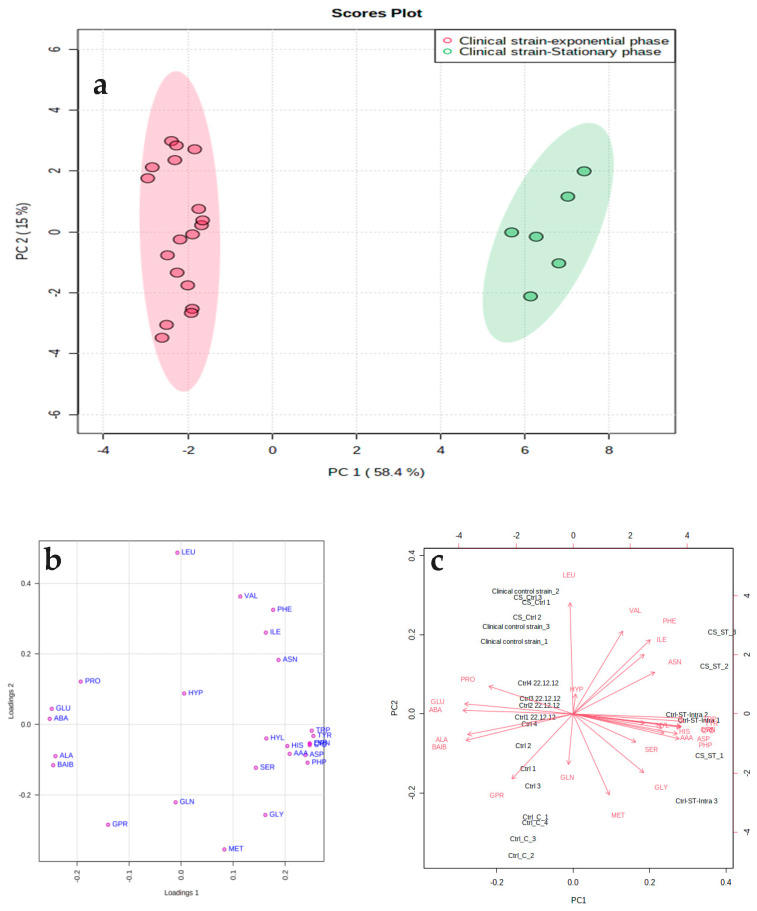
(**a**) Supervised principal component analysis (PCA) plot showing the amino acid patterns of the mid-exponential and stationary phases. The shaded area shows the 73.4% confidence area. The influence of each amino acid within the PCA model is shown in a (**b**) loading plot and (**c**) biplot.

**Figure 4:**
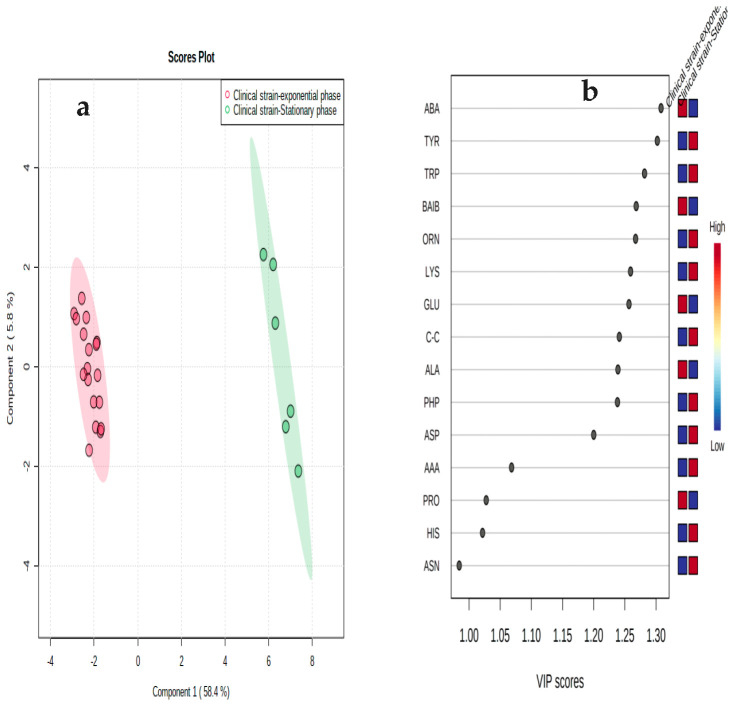
(**a**) Two-dimensional PLS-DA of *S. aureus* amino acids from the exponential and stationary phases. The plots indicate a separation between the mid-exponential phase and the stationary phase. (**b**) VIP plot showing the top 15 amino acids that significantly contributed to the separation between the two growth phases. Boxes on the right indicate the abundance of each amino acid from the mid-exponential and stationary cultures.

The PCA model revealed that the amino acid metabolite patterns of the mid-exponential and stationary phases were significantly different (Figure 3). The variance values were 58.4% for PC1 compared with 15% for PC2. The PCA plot shows that the replicates of the cells harvested at the mid-exponential phase were tightly grouped and very different to the equivalent profile sets measured at the stationary phase. Amino acid data derived from the exponential phase were positioned on the negative side of PC1, while those analyzed at the stationary phase were positioned on the positive side of PC2. The loading plot indicates the key amino acids that significantly contributed to the difference between the two growth phases, and the biplot indicates both the amino acids and replicates of both phases in the PCA model. PLS-DA was applied to identify amino acids that significantly contributed to the classification through the variable importance in projection (VIP) (Figure 4). The model of PLS-DA was validated through cross-validation including the R2 and Q2 (i.e., R2 = 0.99 and Q2 = 0.98) values, which indicate the predictive ability of the model and thus ensure the quality of the model to avoid the risk of over-fitting. The PLS-DA model used 64% of the data; the variance values were 58.4% for Component 1 compared with 5.2% for Component 2. This analysis demonstrated that the samples were closely grouped on the basis of the growth phase, demonstrating high reproducibility within samples and with characteristic amino acid profile compositions for the different growth stages. The amino acids with a VIP score value above 1 and a *p* < 0.05 were considered as important for allowing discrimination between the two phases. Amino acids were ranked according to their VIP scores in the PLS-DA model. These data support the hypothesis that the clinical strain differs in its metabolic response to alterations in environmental conditions where nutrients are limiting and waste products accumulate in the growth medium.

**Figure 5:**
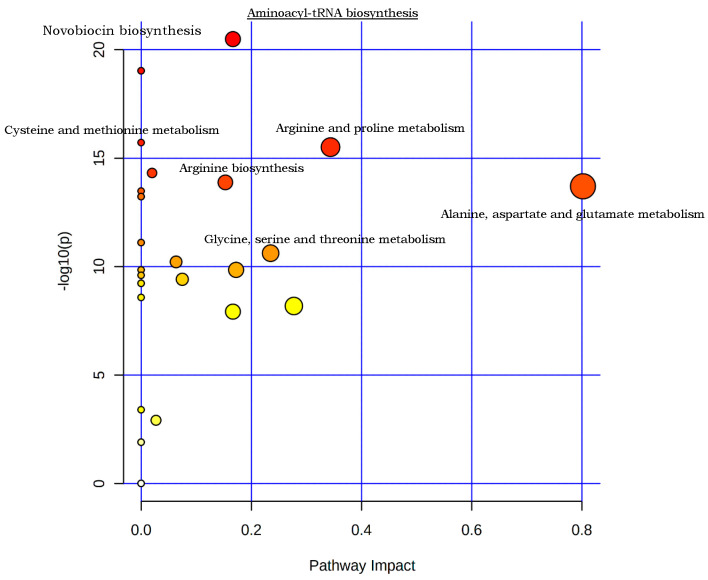
Metabolic pathway investigation of amino acid data extracted from exponential and stationary phase cultures. Each circle is representative of a metabolic pathway. Circles with a red color indicate more significant changes in the amino acid metabolites in the corresponding pathway. The circle size resembles the pathway impact score and is associated with the number of metabolites involved in that metabolic pathway.

Metabolic pathway analysis was performed using the amino acid concentration values to identify variations in the amino acids involved in the same biological pathways. Pathway topology analysis was implemented to detect the particular impact of an amino acid on a particular pathway. All metabolic pathways are presented as nodes (Figure 5). Darker node colors specify the most significant variations in the amino acid metabolites in the corresponding pathway. The size of the nodes represents the pathway impact score and is correlated with the number of amino acid metabolites involved in that pathway (hit numbers). As depicted in Figure 5, twenty three metabolic pathways were identified, and those that had an impact of ≤5 and/or statistical significance above −log (*p*) = 10 were measured as the most significant. The most altered metabolic pathways between the mid-exponential and stationary phases were aminoacyl-tRNA biosynthesis; tyrosine metabolism; novobiocin biosynthesis; taurine and hypotaurine metabolism; arginine and proline metabolism; phenylalanine, tyrosine and tryptophan biosynthesis; arginine biosynthesis; alanine, aspartate and glutamate metabolism; lysine biosynthesis; and D-arginine and D-ornithine metabolism. The pathways were classified according to the total number of metabolites found to be involved the pathway according to the KEGG database, the analyzed amino acids present in the pathway and the *p*-value.

## 4. Discussion

The outcomes of this study show that a clinical isolate of *S. aureus* induced substantial alterations in cytoplasmic amino acid composition during the transition between the mid-exponential and stationary phases. The alterations observed in the amino acids in the current study were in response to the transition in the altered conditions as the cells progressed from the mid-exponential phase to the stationary phase, which included nutrient limitations, the accumulation of metabolic waste products, and pH changes. These consequences support the premise that bacterial cells rapidly adjust to alterations in the environmental conditions by adapting amino acid metabolites to accomplish the best metabolic homeostasis for survival. 

The evaluation of the cytoplasmic amino acid patterns of the stationary phase revealed a remarkable increase in the total amino acid pool, suggesting that the bacterium adjusts its amino acid pool to achieve optimal metabolism related to the changes in the growth media, or perhaps has a storage function. Our earlier publication demonstrated that stationary phase cultures of a clinical isolate of *S. aureus* take up more amino acids from the growth media in comparison with those grown to the mid-exponential phase [20]. This could also be a strategy to enhance protein production, as it has been shown that protein production in the stationary phase was almost 121% higher in comparison with the cells analyzed at the exponential phase [21]. *Escherichia coli* cells in the stationary phase kept producing proteins at a constant rate for some days [6]. It has been demonstrated that protein production is essential for cells of *S. aureus* under long-term starvation [22]. The production of proteins in the stationary phase may be connected with the requirements of the bacteria to withstand the severe conditions present in that phase, including limited nutrients and higher toxin levels.

The shift from grown cells in the exponential phase to non-growing cells in the stationary phase involves complex modifications in cell wall components, protein synthesis and pH homeostasis. This may explain the high level of cytoplasmic amino acid concentration in the cells harvested from the stationary phase in comparison to those analyzed at the mid-exponential phase. Additionally, the high level of cytoplasmic amino acids in cells grown to stationary phase could be associated with the secreted enzymes and amino acids, which may have a role in biofilm formation and quorum sensing. Our previous work has shown that a number of enzymes and amino acids were secreted into external media during the stationary phase [13,20].

The main amino acids that were increased in the stationary phase were aspartic acid, lysine and histidine. This finding is similar to the results of a previous study showing that the lysine levels increased in *S. aureus* during growth in the stationary phase in response to nutrient limitations. This accumulation of lysine could be essential for maintaining pH equilibrium in the cytoplasm, which could become acidified as a consequence of proton influx [23]. Histidine was present at high levels in the stationary phase, which could be driven by higher uptake from the media, since *S. aureus* is unable to synthesize histidine [20,23,24]. Glutamic acid was the major and second most abundant component of the cytoplasmic pool at the exponential and stationary phases, respectively. This may indicate its crucial function as the key link between nitrogen and carbon metabolism. The higher level of glycine at the stationary phase could be associated with a partial or complete reduction in the biosynthesis of purine/pyrimidine and/or cell wall components, as glycine serves as an essential precursor in both pathways. 

There is a remarkable ability in bacterial cells to detect and respond to alterations in environmental conditions to optimize protein synthesis and hence obtain the ideal metabolic homeostasis [25,26,27]. The exponential phase samples under control conditions were considered as six separate groups of three replicates and the results of both PCA and PLS-DA show that the repetition of the growth environments indicated reproducible metabolic phenotypes at the point of harvesting with respect to cytoplasmic amino acid profiles. The alterations in cytoplasmic composition could represent different phenotypic heterogeneity with a bacterial population. *S. aureus* exhibited a reduction in the cell size in response to starvation [22,28]. On this premise, a hypothesis was proposed to suggest that in altered environments, *S. aureus* in the stationary phase are constantly adapting to stressful environments by forming strategies and establishing distinctive phenotypes to resist the alterations in growth conditions. These extensive adjustments of amino acid metabolites in the stationary phase are considered a required element for the development of cell adaptation. Similarly, responses involving the uptake of amino acids were observed in the stationary phase [20]. 

## 5. Conclusions

The changes in the cytoplasmic amino acid composition of *S. aureus* measured in the stationary phase compared with the mid-exponential phase of growth suggested alterations in homeostasis in response to nutrient availability and the buildup of metabolic waste products in the external medium. Certain alterations in amino acid levels would offer an underlying framework for driving adaptive mechanisms associated with the survival of this bacterium under changing environmental conditions. The elucidation of these survival mechanisms of *S. aureus* could provide new insights for interfering with their capacity for surviving between hosts and establishing new infections.

## Figures and Tables

**Table 1:** The levels of cytoplasmic amino acids (nmoles mg^−1^ dry cell mass) extracted from cells grown to the mid-exponential and stationary phases of growth.

Amino Acid	Mid-Exponential Phase	Stationary Phase
Alanine	14.03 ± 5.09	3.38 ± 0.40 *
Glycine	0.97 ± 0.38	4.55 ± 2.12 *
α-Aminobutyric acid	2.13 ± 0.56	0 ± 0 *
Valine	2.30 ± 1.21	6.46 ± 3.29
ß-Aminoisobutyric acid	7.23 ± 1.47	1.62 ± 0.22 *
Leucine	2.36 ± 2.57	2.23 ± 1.31
Allo-isoleucine	0 ± 0	0.25 ± 0.20
Isoleucine	0.25 ± 0.26	1.76 ± 1.13 *
Serine	0 ± 0	1.23 ± 0.33
Proline	16.92 ± 3.98	10.83 ± 7.42 *
Asparagine	1.20 ± 0.71	4.84 ± 1.07 *
Aspartic acid	68.38 ± 15.71	180.45 ± 35.67
Methionine	2.68 ± 0.68	6.14 ± 2.00
4-Hydroxyproline	0.30 ± 0.25	0.47 ± 0.31
Glutamic acid	110.95 ± 34.9	51.94 ± 15.20 *
Phenylalanine	1.15 ± 0.45	3.26 ± 1.31
α-Aminoadipic acid	0 ± 0	3.31 ± 1.90 *
Glutamine	0.66 ± 0.38	2.11 ± 2.99
Ornithine	0.60 ± 0.11	5.53 ± 1.25 *
Glycine-proline	0.58 ± 0.32	0.26 ± 0.29
Lysine	2.98 ± 1.00	67.22 ± 14.29 *
Histidine	7.14 ± 2.82	37.54 ± 11.09 *
Hydroxylysine	0 ± 0	2.87 ± 2.96 *
Tyrosine	0.41 ± 0.10	5.47 ± 0.78 *
Proline-hydroxyproline (dipeptide)	0.02 ± 0.05	1.85 ± 0.44 *
Tryptophan	0.01 ± 0.02	0.99 ± 0.33 *
Cystathionine	0.30 ± 0.23	0.00 ± 0.00
Cystine	0 ± 0	0.42 ± 0.22 *
Total amino acids	243.57 ± 62.1	407.01 ± 47.9 *

* indicates amino acids that were significantly altered.

## Data Availability

Not applicable.
